# Dynamic Bayesian networks for prediction of health status and treatment effect in patients with chronic lymphocytic leukemia

**DOI:** 10.1038/s41598-022-05813-8

**Published:** 2022-02-02

**Authors:** Piotr Ladyzynski, Maria Molik, Piotr Foltynski

**Affiliations:** Nalecz Institute of Biocybernetics and Biomedical Engineering, Polish Academy of Sciences, 4 Trojdena street, 02-109 Warsaw, Poland

**Keywords:** Biomedical engineering, Translational research

## Abstract

Chronic lymphocytic leukemia (CLL) is the most common blood cancer in adults. The course of CLL and patients' response to treatment are varied. This variability makes it difficult to select the most appropriate treatment regimen and predict the progression of the disease. This work was aimed at developing and validating dynamic Bayesian networks (DBNs) to predict changes of the health status of patients with CLL and progression of the disease over time. Two DBNs were developed and implemented i.e. Health Status Network (HSN) and Treatment Effect Network (TEN). Based on the literature data and expert knowledge we identified relationships linking the most important factors influencing the health status and treatment effects in patients with CLL. The developed networks, and in particular TEN, were able to predict probability of survival in patients with CLL, which was in line with the survival data collected in large medical registries. The networks can be used to personalize the predictions, taking into account a priori knowledge concerning a particular patient with CLL. The proposed approach can serve as a basis for the development of artificial intelligence systems that facilitate the choice of treatment that maximizes the chances of survival in patients with CLL.

## Introduction

Chronic lymphocytic leukemia (CLL) is a non-Hodgkin lymphoma and the most common blood cancer in adults^1^. The standardized age-adjusted mean incidence of CLL in years 2013–2017 was equal to 5.0 per 100,000 residents in USA per year^2^. The incidence of CLL in Western countries is similar to that of USA, but in Asian countries (i.e. China, Japan) CLL is extremely rare, and it is estimated to comprise only 10% of all leukemias. The incidence of CLL increases with age. It is rarely diagnosed in people under the age of 40 years. The median age at diagnosis is between 67 and 72 years^3–5^. As the incidence rate rises with age, the prevalence of CLL is expected to keep increasing in coming years due to the society aging.

The disease is more common in men than women with the incidence ratio of 1.7:1.0^6^. Median overall survival has been estimated to be 10 years, but survival of individual patients may vary from months to decades^3^.

CLL is a lymphoproliferative disorder characterized by the clonal proliferation and progressive accumulation of morphologically mature B lymphocytes in the bone marrow, blood, lymphatic nodes and spleen^4^. CLL is diagnosed when lymphocytosis lasts for at least 3 months and the number of lymphocytes is above 5 × 10^9^ per liter^7–9^. The clonality of circulating B lymphocytes must be confirmed by flow cytometry by checking the levels of CD19 + , CD20 + , CD5 + , CD23 + antigens and the presence of λ and κ light chains.

In the majority of patients, i.e. 70–80%, there are no clinical symptoms of the disease at the time of diagnosis of CLL. Moreover, more than 30% of patients with CLL never require treatment and die from causes other than CLL.

A few studies have demonstrated that initiating treatment in the early stage CLL has no effect on survival^7–9^. Therefore, an early-intervention therapy with anti-leukemia drugs, including the Bruton tyrosine kinase inhibitors (BTKi) or B-cell lymphoma 2 (BCL2) antagonists, alone or in combination with monoclonal antibodies, currently is not recommended^2^. In general practice, patients with asymptomatic early-stage disease should be monitored without therapy, applying so called “watch and wait” strategy, unless they have evidence of disease progression or disease-related symptoms.

The decision about the treatment is the resultant of the CLL stage, the presence of symptoms of the disease that impede the functioning of the patient, prognostic factors and disease activity^10,11^. According to iwCLL guidelines symptomatic or active disease is indicated if at least one of the following criteria is met: (1) evidence of progressive marrow failure as manifested by the development or worsening of anemia and/or thrombocytopenia; (2) massive or progressive or symptomatic splenomegaly; (3) massive nodes (the largest diameter ≥ 10 cm) or progressive or symptomatic lymphadenopathy; (4) progressive lymphocytosis with an increase of ≥ 50% over a 2-month period, or lymphocyte doubling time of less than 6 months; (5) autoimmune complications including anemia or thrombocytopenia poorly responsive to corticosteroids; (6) symptomatic or functional extranodal involvement, e.g. skin, kidney, lung, spine; (7) disease-related symptoms such as: unintentional weight loss ≥ 10% within the previous 6 months, significant fatigue, fevers 38.0 °C (≥ 100.5°F) for 2 or more weeks without evidence of infection or night sweats for 1 month or more without evidence of infection^2^.

The effectiveness of treatment is assessed using indicators such as progression-free survival (PFS) and overall survival (OS)^12^. The result of treatment, according to the criteria developed by the National Cancer Institute Working Group, may be: complete remission (CR), partial remission (PR), stable disease (SD) or progressive disease (PD). The sum of CR and PR is defined as the overall response rate (ORR) and it determines the percentage of patients who responded to the treatment^13^. Recently, the assessment of minimal residual disease (MRD) is an increasingly important category of response assessment, resulting in four different response categories^2,4^.

Until recently, the goal of CLL treatment was to keep lymphocytosis under control and to eliminate general symptoms. Currently, with the introduction of new methods of therapy, the goal, especially in younger people, should be to achieve CR, extend PFS and extend the OS, as well as improve the quality of life with maintaining professional activity^14–16^.

People with CLL commonly receive multiple treatments over the course of their disease, such as chemotherapy, radiation therapy, biologic therapy, immunomodulating agents and other oral oncology agents, and allogenic stem cell transplantation. Currently, several CLL treatments are available. Monotherapy with alkylating agents has been considered as an initial, first-line therapy for CLL for a few decades, with chlorambucil being a “gold standard” treatment option^17^. Today, still chlorambucil (CLB) monotherapy may be considered as an inexpensive option in elderly or unfit patients^4^. Monotherapy with purine analogues such as fludarabine, pentostatin and cladribine is also used in CLL. Another treatment option is the use of monoclonal antibodies such as anti-CD20 antibodies (i.e. rituximab, ofatumumab and obinutuzumab) or a monoclonal antibody against CD52 antigen (i.e. alemtuzumab). Recently a few other treatment modalities started to be introduced and tested, including agents targeting the signaling in CLL cells and their environment (i.e. idelalisib, ibrutinib, acalabrutinib, lenalidomid), inhibitors of B-cell lymphoma 2 (i.e. venetoclax, providing a new therapeutic option for very poor prognosis population), programmed death 1 (PD-1) blocking antibodies (i.e. pembrolizumab) and autologous T cells modified using a lentiviral vector expressing a chimeric antigen receptor (CAR) with specificity for the B-cell antigen CD19, coupled with CD137 and CD3-zeta signaling domains^4^. The combined use of different treatment options often leads to improvement of the response to and/or outcome of such a combination therapy in comparison with monotherapy. In younger patients resistant to purine analogs or those with unfavorable prognosis, hematopoietic cell transplantation with curative intent is also used^18^.

The course of CLL and patients' response to treatment are varied, which is reflected in the available results of the randomized controlled trials (RCTs) comparing the effectiveness of selected treatment options. This variability makes it difficult to select the most appropriate treatment regimen and predict the progression of the disease for a given patient. When deciding on initiating the treatment and selecting the treatment regimen, the following parameters should be taken into consideration: the clinical stage of the disease, the general condition or the fitness and comorbidities of the patient, changes in the blood and marrow lymphocytes phenotype, prognostic factors, history of the treatment (e.g. first *vs.* second line, response *vs.* nonresponse to the last treatment), and family history of the disease. It should be also taken into consideration that during the relapse of the disease the effectiveness of subsequent treatment lines is lower due to the selection of clones of cells resistant to the previously used drugs. That is why choosing the right treatment is extremely difficult and needs to be personalized.

This work is aimed at developing and validating an artificial intelligence system using the dynamic Bayesian network (DBN) framework to predict changes of the health status of patients with CLL and progression of the disease over time to create a basis for personalized treatment planning and maximizing the survival time.

Bayesian networks, thanks to their properties, i.e. simple structure and clear reflection of knowledge about causal dependencies and by combining expert knowledge with data are convenient tools for developing systems supporting medical diagnosis and treatment^19^. In general, Bayesian methods are particularly suited for capturing and processing uncertainty. They have been used in biomedical engineering and health-care for a few decades and have been applied, among others, in handling inexact knowledge associated with the risk prediction^20^, eliciting subjective opinion regarding CLL treatment^21,22^, establishing diagnosis of CLL with the use of genomic^23–25^ or flow cytometry^26^ data, and selecting optimal treatment in patients with CLL^27–31^.

Several of the above-mentioned studies investigated treatment effect in patients with CLL. However, none aimed to use the domain literature, medical recommendations and experts’ knowledge to select the most important parameters influencing the health status and treatment outcomes, and to quantify the strength of the interaction between these factors, in terms of conditional probabilities, to be able to predict the posteriori probability of temporal transition of these parameters between possible states utilizing a priori knowledge.

## Results

### Health status network

Due to complexity of the problem, the first DBN that we developed, which was called Health Status Network (HSN), was aimed at predicting the health status of patients with CLL, assuming that each patient was treated in accordance with the best medical practice, reflected in the results presented in the available medical literature. This means that in HSN there are no nodes directly referring to the treatment of the patient, but the effect of the treatment indirectly influences changes of other nodes that are represented in the network. This approach made it possible to simplify the network structure, reduce the number of probabilities necessary to define conditional probability tables (CPTs) in individual network nodes, shortened the calculation time and allowed to determine, which nodes have the largest impact on the patient survival. Staring from the basic network, which is described in “Methods” section, modeling a health status of the patient with CLL at the time of diagnosis with the use of 3 nodes: *Health*, *Disease* and *Complications* we found, based on the literature review, that *Health* node is affected by: general condition of the patient, numerically expressed according to the Eastern Cooperative Oncology Group Performance Status (ECOG scale), age and sex of the patient, family history of CLL and survival. We concluded that *Complications* node is affected by parameters that may worsen the patient's condition, but are not directly related to the disease, i.e. concomitant diseases, other cancers and infections.

We found that *Disease* node is affected by parameters directly related to CLL, i.e. the stage of CLL, the phenotype of blood and marrow lymphocytes, cytogenetic aberrations, hematological tests, blood serum tests, abdominal palpation and the treatment applied, i.e. type of therapy, date of treatment initiation, prescribed drugs, the reason for discontinuation of treatment and the treatment result.

**Figure 1:**
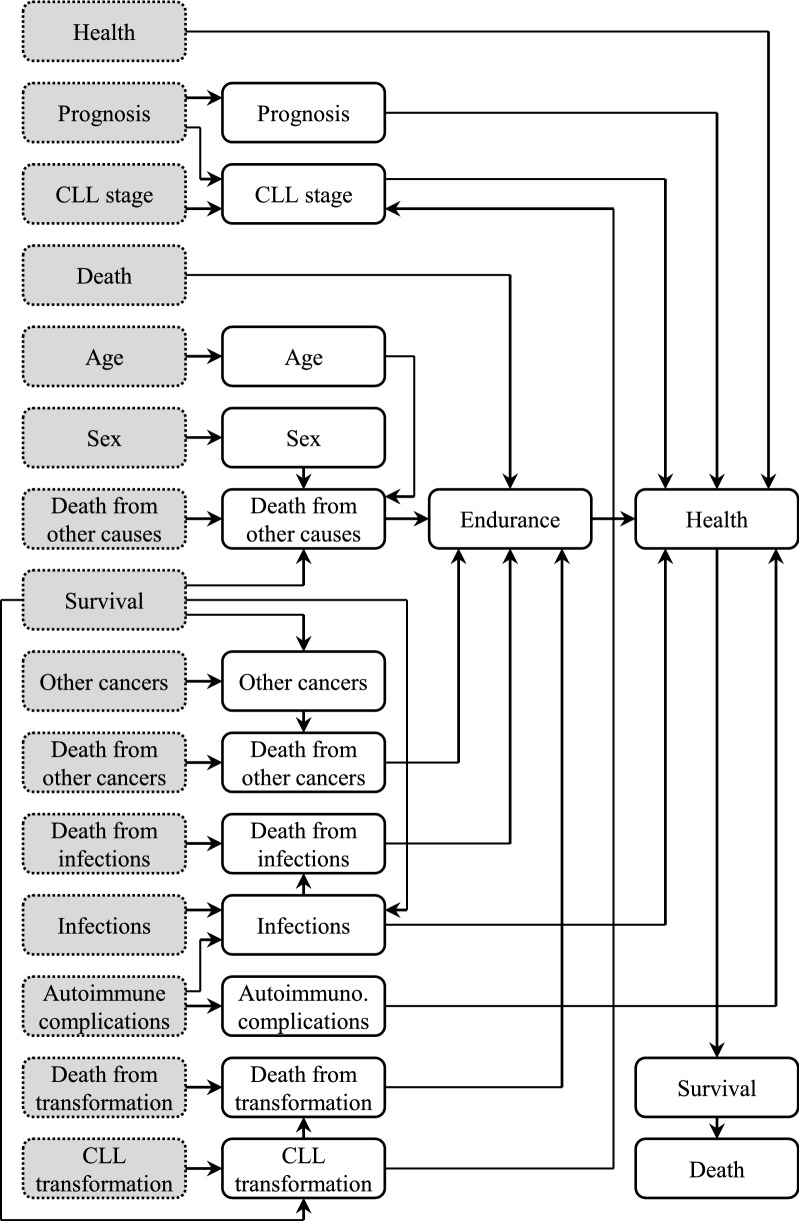
Diagram of the Health Status Network (HSN). The network nodes at moments *t-1* have gray background and dotted borders, and the nodes at moment *t* have white background and solid borders.

Altogether, the HSN consists of 16 nodes at the initial moment *t* = *0* and subsequent 16 nodes at the next considered moment. The time slice is equal to 6 months. The diagram of HSN is shown in Fig. 1. Two nodes (i.e. *Prognosis* and *Stage of CLL*) may take one of three values and the remaining 14 nodes are bi-valued. Table 1 shows description of all 16 nodes.

**Table 1:** Nodes of the health status network.

Node name	Node description	Node values
Age	Patient’s age (up to 65 years or above)	≤ 65 years, > 65 years
Autoimmune complications	Complications related to hematologic disorders, i.e. thrombocytopenia and anemia	Yes, no
CLL stage	CLL stage according to the Rai staging system^32^	0, I–II, III–IV
CLL transformation	Transformation of CLL into an aggressive form of leukemia, mainly Richter syndrome	Yes, no
Death	Patient died (regardless of the cause of death)—opposite to *Survival*	Yes, no
Death from CLL transformation	Patient died as a result of transformation of CLL	Yes, no
Death from infections	Patient died as a result of infections or is alive	Yes, no
Death from other cancers	Patient died as a result of cancers other than CLL	Yes, no
Death from other causes	Patient died due to reasons other than CLL, CLL transformation, infections or other cancers	Yes, no
Endurance	Patient is alive or died due to reasons other than CLL	Yes, no
Health	Patient’s general health condition according to ECOG Scale of Performance Status^33^	ECOG 0–2, ECOG 3–4
Infections	Patient suffers from bacterial, viral or fungal infections	Yes, no
Other cancers	Patient has other cancers besides CLL, e.g. colorectal cancer	Yes, no
Prognosis	Synthetic prognosis based on analysis of cytogenetic parameters	Good, intermediate, poor
Sex	Patient’s gender	Female, male
Survival	Patient is alive or died (regardless of the cause of death)—opposite to *Death*	Yes, no

An example of the CPT content of one of the nodes of HSN for which CPT was determined on the basis of the expert’s knowledge, is shown in Table 2.

**Table 2:** Conditional probability table for *CLL stage* node in the health status network.

Prognosis (t-1)	CLL stage (t-1)	CLL transformation (t)	CLL stage (t)		
			III–IV	I–II	0
Good	III–IV	Yes	1.00	0.00	0.00
Intermediate	III–IV	Yes	1.00	0.00	0.00
Poor	III–IV	Yes	1.00	0.00	0.00
Good	I–II	Yes	1.00	0.00	0.00
Intermediate	I–II	Yes	1.00	0.00	0.00
Poor	I–II	Yes	1.00	0.00	0.00
Good	0	Yes	1.00	0.00	0.00
Intermediate	0	Yes	1.00	0.00	0.00
Poor	0	Yes	1.00	0.00	0.00
Good	III–IV	No	0.90	0.10	0.00
Intermediate	III–IV	No	0.95	0.05	0.00
Poor	III–IV	No	1.00	0.00	0.00
Good	I–II	No	0.00	0.80	0.20
Intermediate	I–II	No	0.05	0.90	0.05
Poor	I–II	No	0.10	0.90	0.00
Good	0	No	0.00	0.00	1.00
Intermediate	0	No	0.00	0.40	0.60
Poor	0	No	0.05	0.95	0.00

### Treatment effect network

The Health Status Network was the first stage of the work leading to design of the Treatment Effect Network (TEN) that predicts treatment outcome and survival of the patient based on her / his health status and the treatment administered.

The developed TEN is a DBN consisting of 18 nodes, which can take from two (e.g. *Sex* or *Survival* node) to four (e.g. *Treatment* or *Previous treatment* node) discrete values.

**Figure 2:**
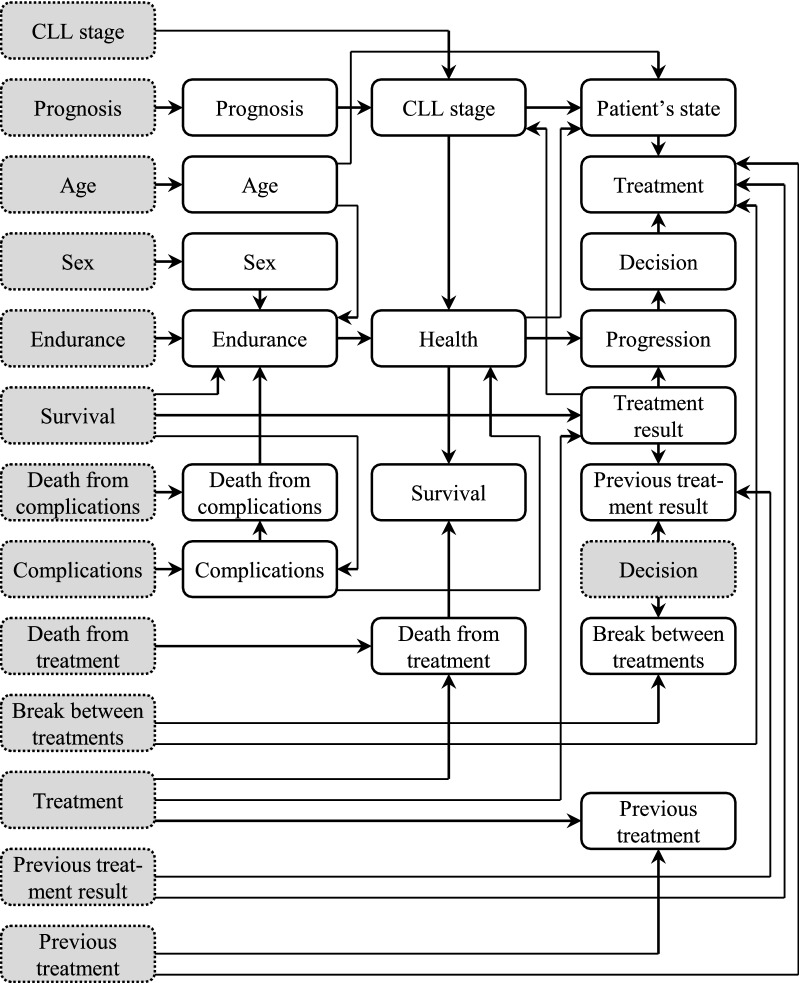
Diagram of the Treatment Effect Network (TEN). The network nodes at moments *t-1* have gray background and dotted borders, and the nodes at moment *t* have white background and solid borders.

The diagram of TEN showing links between individual nodes is illustrated in Fig. 2 and the description of all the nodes is provided in Table 3. The CPT content of *Treatment* node of TEN is shown in Table 4.

**Table 3:** Nodes of the treatment effect network.

Node name	Node description	Node values
Age	Patient’s age (up to 65 years or above)	≤ 65 years, > 65 years
Break between treatments	Time that has elapsed after the previous treatment was applied	≤ 6 months, > 6 months
CLL stage	CLL stage according to the Rai staging system^32^	0, I–II, II–IV
Complications	Other cancers, infections, autoimmune complications, Richter syndrome	Yes, no
Death from complications	Patient died as a result of complications	Yes, no
Death from treatment	Patient died as a result of treatment complications	Yes, no
Decision	Results of analysis regarding necessity of treatment initiation	Treatment, watch and wait, death
Endurance	Patient is alive or died due to reasons other than CLL and treatment complications	Yes, no
Health	Patient’s general health condition according to ECOG Scale of Performance Status^33^	ECOG 0–2, ECOG 3–4
Patient’s state	Parameter which decides whether more aggressive treatments are applicable	Good, poor
Previous treatment	Previous-line treatment (the same values as in *Treatment* node)	None, AA, PA, MA^a^
Previous treatment result	Result of the previous-line treatment (the same values as in *Treatment result* node)	CR + PR, SD, PD^b^, death
Prognosis	Synthetic prognosis based on analysis of cytogenetic parameters	Good, intermediate, poor
Progression	The applied treatment has resulted in progression of the disease or not	Yes, no
Sex	Patient’s gender	Female, male
Survival	Patient is alive or died (regardless of the cause of death)	Yes, no
Treatment	Current-line treatment: alkylating agents (AA), purine analogs (PA) or monoclonal antibodies (MA)	None, AA, PA, MA^a^
Treatment result	Result of the current-line treatment: complete or partial remission (CR + PR), stabilization (SD), progression (PD)	CR + PR, SD, PD^b^, death

^a^*AA* alkylating agents, *PA* purine analogs, *MA* monoclonal antibodies.

^b^*CR + PR* complete + partial remission, *SD* stable disease, *PD* progressive disease.

**Table 4:** Conditional probability table for the *Treatment result* node in the Treatment Effect Network.

Treatment (t-1)	Survival (t-1)	Treatment result^a^ (t)			
		CR + PR	SD	PD	Death
None	Yes	0.01	0.95	0.04	0.00
Alkylating agents	Yes	0.60	0.21	0.19	0.00
Purine analogs	Yes	0.75	0.10	0.15	0.00
Monoclonal antibodies	Yes	0.72	0.25	0.03	0.00
None	No	0.00	0.00	0.00	1.00
Alkylating agents	No	0.00	0.00	0.00	1.00
Purine analogs	No	0.00	0.00	0.00	1.00
Monoclonal antibodies	No	0.00	0.00	0.00	1.00

^a^*CR + PR* complete + partial remission, *SD* stable disease, *PD* progressive disease.

### Implementation and testing

Implementation of HSN and TEN was done using the Bayes Net Toolbox (BNT) software package^34^, which is an addition to the Matlab system. For inference we used *jtree_dbn_inf_engine*, which applies the junction tree algorithm to pairs of neighboring slices at a time.

After defining CPTs of HSN and TEN, performance of the networks was tested. For each node, results of simulations using a priori probabilities reflect results of an average patient with CLL. For some nodes, e.g. *Survival*, the results of the conducted simulations can be compared with the clinical information from the cancer registries (CRs) collecting data of a large number of patients with CLL who underwent different types of treatment.

In case of both networks for such a comparison we used the survival data from the US and Europe. In the US the survival results of patients with CLL come from the Surveillance, Epidemiology, and End Results (SEER) Program (SEER*Stat Database), which contains the data of 18 CRs covering 28% of the US population^35,36^.

From Europe we used the pooled survival data of patients with CLL and B cell small lymphocytic lymphoma (SBLL) from the EUROCARE-5 database, which collected the data from 99 CRs covering 50% of population of 29 European countries^37,38^. In both databases, i.e. SEER*Stat and EUROCARE-5, we used the survival data of patients who were diagnosed in years 2000–2007. EUROCARE-5 is freely available and the access to SEER*Stat is granted upon registration. It is noteworthy that these databases provide the survival data with limited tools for subgroup analysis (e.g. male *vs.* female), which are hardly usable to learn CPTs in nodes of the network.

Output of other selected nodes of HSN and TEN were also verified using the literature data. In the case of HSN probabilities of transformation of CLL into an aggressive form of leukemia, mainly the Richter syndrome, predicted using HSN and reported by Perikh et al.^39^ were compared. Whereas in the case of TEN we evaluated feasibility of the network in predicting effectiveness of selected treatment options in comparison with the available results of clinical trials. Two such comparisons were made. They concerned survival of the treatment-naïve patients with CLL in whom the first drug used was one of the alkylating agents^40–42^ or purine analogues^40–44^ used as a monotherapy or in combination with other drugs.

An advantage of Bayesian networks is the ability to take into account a priori knowledge in performed simulations by modifying the CPT content of individual network nodes. To illustrate this feature in relation to TEN, the survival curves of three groups of patients with CLL were simulated: (1) all patients, (2) patients with no treatment-related side effects leading to death, (3) patients with complications such as infections, autoimmune complications, other cancers or Richter syndrome during the period from diagnosis of CLL.

### Predictions of the networks vs. clinical results

**Figure 3:**
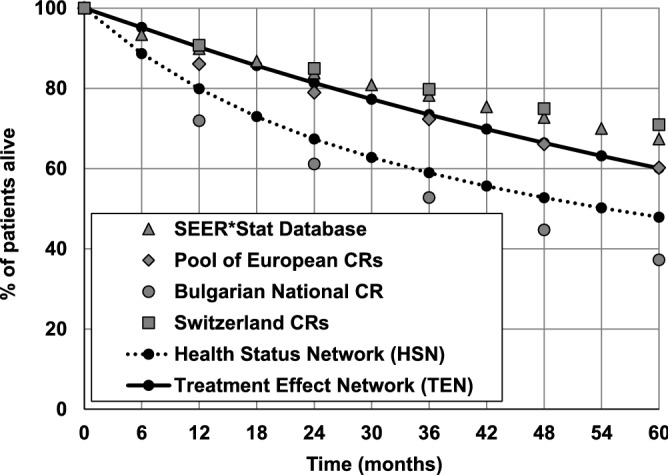
Percentage of patient with CLL alive within 60 months from diagnosis. Survival of patients with CLL predicted by the Health Status Network (HSN) and Treatment Effect Network (TEN) in comparison with results collected in SEER*Stat^35,36^ and EUROCARE-5 databases^37,38^.

In Fig. 3, the survival data predicted by HSN and TEN are compared with 60-months survival results from SEER*Stat and EUROCARE-5 databases in patients who were diagnosed with CLL in years 2000–2007.

In case of the EUROCARE-5, in addition to the pooled survival data, the results from CRs in regions with the highest (i.e. CRs in Switzerland covering 30% of national population: Basel, Geneva, Grisons, St. Gallen, Ticino and Valais) and the lowest (Bulgarian National CR covering 100% of the country population) survival rates are presented. The survival curve predicted using HSN lies below the survival curves, which were obtained using the data collected in EUROCARE-5 and SEER*Stat databases. The mean absolute difference calculated yearly during the first 60 months after CLL diagnosis is equal to 11.4% and 20.9% in comparison with the real-life survivals calculated based on the data in EUROCARE-5 and SEER*Stat database, respectively. Nevertheless, the simulated results lie between the boundaries outlines by the survival data collected in the European regions where the treatment of patients with CLL is the least and the most efficient.

The survival curve predicted using TEN for an average patient with CLL is very close to the real survival curve calculated based on the pool of European CRs. The mean absolute difference is equal to 1.6% when calculated based on 5 survival rates reported yearly for the first 60 months after CLL diagnosis. In case of SEER*Stat database the mean absolute difference is larger, i.e. 7.8%, and TEN underestimates the real-life survival data starting from the second year after CLL diagnosis.

**Figure 4:**
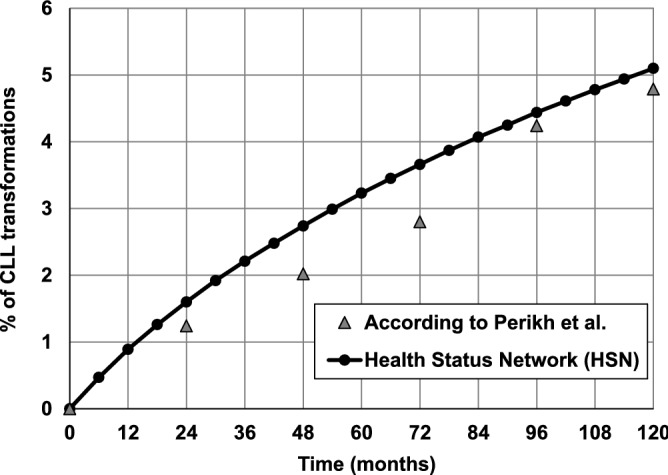
The probability of transformation of CLL into an aggressive form of leukemia within 10 years from the CLL diagnosis. Comparison of the probability predicted by the Health Status Network and reported by Perikh et al.^39^.

In Fig. 4 probability of transformation of CLL into an aggressive form of leukemia predicted using HSN and reported by Perikh et al.^41^ is compared.

The course of changes in the probability of CLL transformation into a more aggressive form obtained during simulation is similar to the course of this probability reported in the literature^41^ over a period of 10 years. The mean absolute difference calculated biannually is equal to 0.49%.

**Figure 5:**
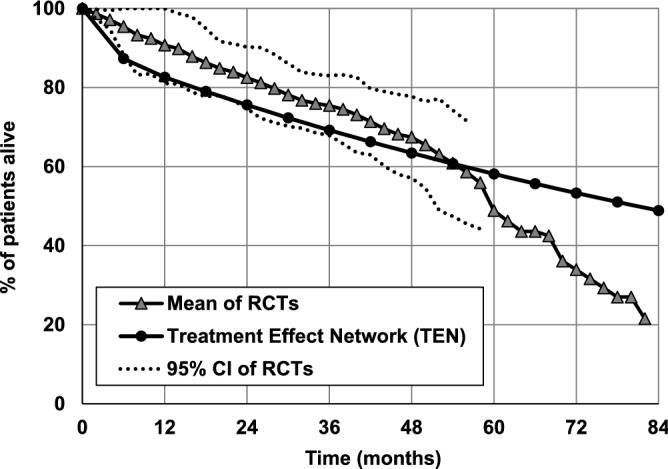
Probability of survival for patients with CLL treated with alkylating agents as the first-line treatment. Comparison of the probability predicted by the Treatment Effect Network and calculated based on results of RCTs^40–42^. The dotted lines indicate 95% confidence intervals (95% CIs).

Figure 5 shows the probability of survival of patients with CLL in whom the first drug used was one of the alkylating agents, predicted using TEN and determined based on the literature data^42–44^.

The simulation results fall within 95% confidence intervals (95% CIs) of the survival results obtained in RCTs. The differences between survival rates predicted by TEN and the mean values calculated using the results of the clinical trials start to increase 60 months after the CLL diagnosis.

**Figure 6:**
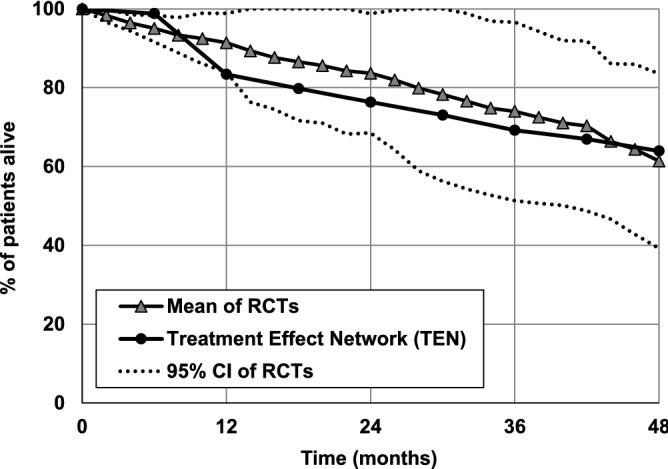
Probability of survival in treatment-naïve patients with CLL treated with purine analogs. Comparison of the probability predicted by the Treatment Effect Network and calculated based on results of RCTs^40–44^. The dotted lines indicate 95% confidence intervals (95% CIs).

In Fig. 6, similar comparison is presented concerning purine analogs used as the first-line treatment as the mono- or combination therapy^42–46^.

The simulation results obtained with the use of TEN are within 95% CIs of to the average survival rates reported in RCTs for 48 months. However, it must be stressed that discrepancies between results of individual clinical trials are high, which is demonstrated by wide 95% CIs.

In Figs. 5 and 6 the data are presented starting from the date of the first treatment, not from the date of CLL diagnosis to reflects the source data from the clinical studies presented on these Figures. These data were obtained by adding the a priori knowledge to the network informing it that a particular treatment should be applied at the beginning of the simulation period. This is an advantage of the Bayesian networks that the initial conditional probability tables can be modified to reflect a priori knowledge in the results of simulations.

**Figure 7:**
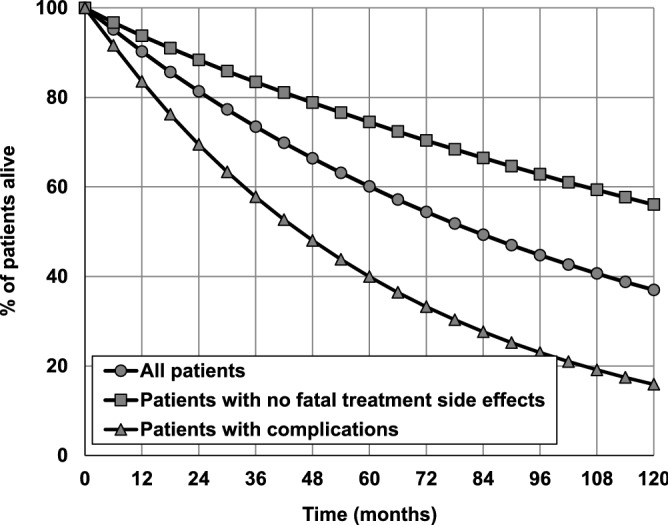
Survival curves of three groups of patients with CLL predicted using the Treatment Effect Network. Comparison of the results predicted in patients with complications, patients with no fatal treatment side effects and all patients.

Figure 7 shows that the probability of surviving 10 years after the diagnosis of CLL is equal to 37% for the average patient, whereas it is 56% for the patient who does not experience fatal treatment side effects, and it is only 16% in patients with complications such as other cancers, infections, autoimmune complications or Richter syndrome. In this example a priori knowledge was included by modifying CPT in *Death after treatment* node for group 2 and *Complications* node for group 3 at any time t. It is also possible to modify CPT in several nodes at the same time at selected moments *t*. Such modifications allow predicting the condition and treatment outcome of more precisely defined groups of patients or even an individual patient, thus, creating a framework for planning the personalized treatment.

## Discussion

In this work two DBNs were developed, i.e. Health Status Network and Treatment Effect Network. The first network—HSN allows predicting, among others, probability of survival, death due to infections, death from other cancers, death from causes not related to CLL and death from transformation of CLL into a more aggressive form of leukemia, in both, the patient described by average values of all the parameters included in the network when no a priori knowledge about a particular patient is available as well as the patient in whom the values of some parameters are known, i.e. when a priori knowledge about the patient can be used to adjust probabilities in CPTs of some nodes in the network. The HSN does not contain nodes directly related to the treatment, but the treatment effect is reflected in values of conditional probabilities in CPTs of nodes, which are present in the network.

The second network—TEN allows to predict probability of survival, death from treatment, death due to complications related to CLL, death due to causes unrelated to CLL, but unlike HSN it contains nodes directly related to the selected treatment option as well as the course and the result of the treatment. As in the case of HSN, also in TEN simulations can be performed both—for the averaged patient and for the specific patients for whom a priori knowledge can be used to modify content of CPTs of particular nodes of the network.

Verification of the developed networks included a comparative analysis of simulation results for selected nodes with the results of clinical trials reported in the medical literature. In comparison with survival probabilities calculated based on the data of tens of thousands of patients gathered in medical databases, i.e. EUROCARE and SEER*Stat the HSN underestimates percentage of patients alive. A few possible reasons exist, which may explain the obtained discrepancies. Firstly, the network structure represents a compromise between the desire to make the most accurate prediction of clinical results on the one hand and the availability of the data that are necessary to determine the structure of the network and the content of CPTs in individual nodes on the other hand. Secondly, the structure of HSN is oversimplified and it does not contain all the variables and interrelations influencing the temporal changes in the health status of patients with CLL. Thirdly, probabilities in CPTs of all the nodes constituting HSN have been set constant whereas one can expect that those probabilities are changing with time. Additionally, in the simulations we used in all nodes CPTs with probabilities not modified by any a priori information reflecting populations whose data were collected in registries or who participated in the clinical trials. It is possible that characteristics of these populations differed from the characteristics reflected in the CPTs of the network. For example, it can be hypothesized that at least some of the cancer centers providing data to the both above-mentioned databases (especially SEER*Stat) are reference centers offering better than average access to modern treatment methods and thus, a survival probability of patients treated in these centers is higher than the mean value modeled in the developed network. If a specific population characteristics is available then this a priori information can be used to modify the content of CPTs to fine-tune predicted survival rates or network response at another selected node to those achieved in a particular country or a particular cancer center. Nevertheless, in some nodes HSN was able to predict respective probabilities with higher accuracy than the overall survival. For example, the probability that CLL is transformed into an aggressive form of leukemia predicted using HSN is in a good agreement with the results reported in the literature. Also the simulation results regarding the probability of death due to transformation of CLL into more aggressive cancers are similar to the data from published clinical trials.

The ability of TEN to correctly predict overall survival of patients with CLL was improved in comparison with HSN although both networks suffers from the same, above-mentioned, weaknesses. Absolut differences of the survival rates predicted using TEN and calculated based on the data available in the EUROCARE-5 database were in average smaller than 2% during 5 years following the CLL diagnosis. In case of the data from the SEER*Stat registry the absolute differences were smaller than 8% in the same period.

The analysis of survival probabilities, when a priori knowledge about the type of the first-line treatment was taken into consideration, demonstrated that the results of simulations using TEN were in agreement with the average results of the available randomized clinical trials, i.e. they were contained inside 95% confidence intervals for a period of approximately 5 years from the application of the treatment for alkylating agents and purine analogs. After this period, the probability of death reported in clinical trials increases significantly whereas the predicted probability is stable, which results in an increase of a difference between the actual and the predicted values. This increase is due, on the one hand, to a small number of patients observed for more than 5 years in clinical trials, and on the other hand, to possible changes in probability of survival over time, which are not correctly reflected in CPTs of the TEN nodes, where all the conditional probabilities are constant in time.

The most important limitation of the presented work is that there are no objective clinical data available to apply machine learning techniques to learn the structure of the networks or calculate the contents of CPTs in its nodes. Consequently, the final networks are truly belief networks reflecting interconnections among particular nodes that were revealed based on the domain literature, medical recommendations and the expert knowledge. As far as content of CPTs is concerned, it is, for example, relatively easy to find the data to define distribution of all cases of CLL among men and women. However, the contents of CPT in *Health* node of TEN, linking the health status of the patient in ECOG scale with *Endurance*, *Complications* (including other cancers, infections, autoimmune complications and Richter syndrome) and CLL stage, must be guesstimated by the medical expert, because such detailed clinical data are not available. Another limiting factor of our approach is related to the fact that building the domain knowledge on CLL treatment takes time. Consequently, we were not able to include in *Treatment* node the emerging treatment options such as BTKi or BCL2 inhibitors, despite the fact that they appeared in the newest recommendations for the first-line treatment in patients with CLL.

Although all the nodes of HSN and TEN are observable (i.e. each node can be set as the output for simulations), the performance of the network can be validated only in those nodes for which there is adequate amount of medical data available. Therefore, we tested the performance of networks mainly on survival data. However, our goal was not to fine-tune the network to get as close to the survival data from the particular registries as possible, but rather develop a network which catches the most important interdependencies between nodes and does not generate response in any node which is contradictory to the literature data. Then, we checked how close such a network is able to reproduce survival data from the available registries. It should be emphasized that to use TEN as an effective tool that facilitates planning of a personalized treatment it is more important that the network is able to properly reflect relative dependencies between nodes and values within the nodes than that it is able to demonstrate high agreement in predicting output of the particular node with real clinical data.

It is noteworthy that the developed DBNs are much more flexible in incorporating patient evidence and answering prognostic queries compared to the proportional hazard models. The last-mentioned are able to model patient-specific covariates that modulate the patient hazard. However, such models cannot provide a causal explanation of how the covariates interact to influence patient survival. Furthermore, assumptions of proportionality, linearity and additivity of the covariates may not be fulfilled.

To the best of our knowledge the presented work constitutes the first attempt to develop dynamic Bayesian networks, which take into account temporal, causal and decision-making characteristics and forecasts the health status and the outcomes of treatment in patients with CLL. The obtained results confirmed feasibility of the presented approach. The results also showed that addition of nodes directly related to the applied treatment improved ability of the Bayesian network to predict survival curves for an average patient with CLL.

In future, the Treatment Effect Network can be further expanded to accommodate more nodes and more values in the existing nodes, in particular those related to different treatment options and prognostic factors, to more accurately reflect clinical results and to take into account new therapies being offered to patients with CLL. However, to be able to use more extensive DBNs the exact inference algorithms, which are implemented in the Bayes Net Toolbox, should be substituted with some approximate algorithms, e.g. approximate particle filtering, which comprises the group of sequential Monte Carlo methods for dynamic state estimation^45^.

In conclusion, we demonstrated that it was possible, based on the literature data and expert knowledge, to design and develop dynamic Bayesian networks trying to quantify relationships linking the most important factors influencing the health status and treatment effects in patients with CLL. The developed networks, and in particular the Treatment Effect Network, were able to effectively estimate probability of survival in patients with CLL. The developed networks can be used as a framework to personalize these predictions by adjusting CPTs, taking into account a priori knowledge concerning a particular patient with CLL. Consequently, the proposed approach can serve as a basis for the development of artificial intelligence systems that facilitate the choice of treatment that provides the best possible effectiveness of therapy and maximizes the chances of survival in patients with CLL.

## Methods

In this work, we use the DBN methodology to predict the health status and treatment effect in patients with CLL. This methodology extends the formalism of standard Bayesian networks, and helps to capture the changing effect of some disease or treatment parameters on other parameters as the disease progresses over time, i.e. has the ability to represent temporal nature of the disease progression and implementation of a medical treatment strategy^46^. Moreover, the DBN framework offers features that are particularly suitable in medical applications, i.e. a causal modeling approach and the ability to learn from patient data. However, the heterogeneity of the course of the disease, its usually long duration, the multitude of treatment options and the fact that new treatments are introduced before the effectiveness of the earlier treatments was fully evaluated, mean that there are no large amounts of high-quality data that could be used to learn the structure and parameters of a particular prognostic model. Therefore, techniques that relay on such raw data and use machine learning (e.g. neural networks, support vector machines, decision-trees or partitioned DBNs) cannot be applied^47^. Therefore, the question arises whether, in such a case, it is possible to build an effective model based on the domain literature data and the expert knowledge. We tried to check it by recreating the way a physician learns how to treat a patient with CLL by studying the literature and asking better experienced colleagues. The DBN framework creates suitable platform to develop and test such a networks. The process of defining the network is not fully systematized, i.e. probably if other researchers had studied the same CLL literature and talk to the same CLL expert physicians, the network they would have created would be different from ours. This is reflected in the alternative name of Bayesian networks which are called belief networks. Yet, this is also similar to the way physicians learn—they all have the same literature and recommendations on disposal, but ultimately each of them may have a different opinion on the treatment of a particular case. Besides, learning of the structure of the model from the data has also a serious limitation, i.e. what can be learned is limited to what is represented in the data collected in a particular database. Therefore, neither in the case of learning the network structure from the data nor in the case of designing the network structure based on the literature and the expert knowledge one can ensure that all the relevant nodes are included in the network or, contrary, that some non-relevant relations are captured in the network.

In the case of CLL, there is a wealth of literature and the expert knowledge to learn from, including results of RCTs comparing effectiveness of different treatment options. This information is usually available in aggregated/averaged form, without sharing results of individual subjects.

The DBN models a multidimensional variable ***X*** over a time horizon [0, *T*], where the variable at the present time *t* can be calculated directly from parent variables at time *t-1* and *t*. Therefore, the transitions of DBN can be expressed as a conditional Bayesian network over ***X***^*(t)*^ conditioned on ***X***^*(t-1)*^, i.e. a directed acyclic graph *G* with node set ***X***^*(t-1)*^* ∪ ****X***^*(t)*^, which defines a conditional distribution according to Eq. (1).

1 $$P\left({{\varvec{X}}}^{(t)}|{{\varvec{X}}}^{(t-1)}\right)=\prod_{i=1}^{n}P\left({X}_{i}^{(t)}|{\pi }_{G}({X}_{i})\right)$$

where: *π*_*G*_*(X*_*i*_*)* ⊆ ***X***^*(t-1)*^* ∪ ****X***^*(t)*^ denotes the parent of *X*_*i*_ in *G.*

We assume that the DBN is time invariant, and thus it can be defined in terms of a priori network ${\mathscr{B}}_0$, i.e. the structure of the network at time *t* = *0* and a transient network determining the changes that individual nodes are subject to, and their interrelationships as the modeled process progresses from time *t-1* to time *t*. DBN is defined as a dynamic system over *[****X***^*(0)*^*,…, ****X***^*(T)*^*]* with the initial distribution, which is given by a Bayesian network ${\mathscr{B}}_0$ over ***X***^*(0)*^ and transition distribution is given by a transient Bayesian network ${\mathscr{B}}$_→_ over ***X***^*(t)*^ conditioned on ***X***^*(t-1)*^. Hence, the unrolled DBN is expressed according to Eq. (2).

2 $$P({{\varvec{X}}}^{(0:T)})=\prod_{i=1}^{n}P\left({X}_{i}^{(0)}|{\pi }_{G}({X}_{i},{\mathcal{B}}_{0})\right)\prod_{t=1}^{T}\prod_{i=1}^{n}P\left({X}_{i}^{(t)}|{\pi }_{G}({X}_{i},{\mathcal{B}}_{\to })\right)$$

We designed networks for patients with CLL based on: state-of-the-art data on progression of CLL and effectiveness of different treatment options, recommendations concerning the diagnosis and treatment of CLL, anonymized data of patients with CLL collected in a BIAL registry^48^, and medical experts’ knowledge. The work was performed in accordance with relevant guidelines and regulations. We have not conducted any experiments involving human subjects, but we used the aggregated and anonymous data concerning people with CLL gathered from the publicly or on request available databases / registries and scientific papers.

**Figure 8:**
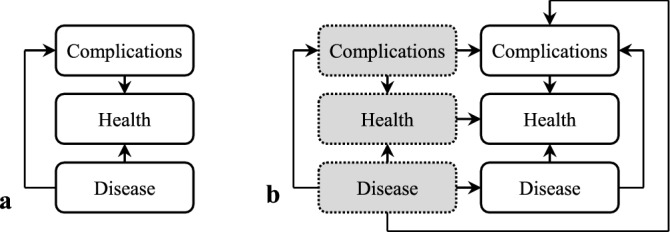
Simple Bayesian network modeling the health condition. (**a**) the static network defining the initial state at *t* = *0* and (**b**) the dynamic network defining the state of nodes in two consecutive time slices, i.e. *t-1* (gray background and dotted border) and t (white background and solid border) and transition between them (solid lines with arrows).

The design of networks started by defining the basic variables (nodes) characterizing the diagnosis and treatment of CLL and the interrelations between them^46^. In the most simplified form, the network modeling health status of the patient with CLL at the time of diagnosis, i.e. at time *t* = *0*, consists of 3 nodes: *Health* describing all variables related to health condition, *Disease* including variables resulting from CLL and *Complications* summarizing variables associated with any complications accompanying CLL, also those unrelated to CLL (Fig. 8a). *Disease* and *Complications* influence *Health*, hence edges connecting these nodes are present in Fig. 8a. In addition, *Disease* can affect *Complications*.

In order to forecast changes in the status of individual nodes over time, it is necessary to define relations between the values of nodes in two consecutive considered times (i.e. *t-1* and *t*) (Fig. 8b).

In Fig. 8b we assumed that *Complications* occurring at time *t* can be affected by the state of *Complications* in the previous moment (*t-1*) and the state of *Disease* in both—the previous and the current moments. In the subsequent stages of network design, we detailed its structure, using information found in the sources listed above, which define how diagnostic parameters, treatment that was applied and additional factors influence the nodes already present in it or how the nodes present in the network should be divided.

The value of a given node at the time *t* depends only on the value of those nodes forming the network, i.e. predecessors, at moments *t-1* and *t*, which are connected to it and the connection runs towards the considered node. In each node a CPT has been defined that makes it possible to calculate the probability that a given node will adopt one from possible values depending on the value of its predecessors.

Such CPTs were created for each network node separately for the moment *t* = *0*, i.e. the moment of CLL diagnosis and each subsequent moment assuming a 6-month period elapsing between the times *t-1* and *t*.

To illustrate the development of the network, let’s consider the *Death from other cancers* node, which is bi-valued, i.e. can take values *yes* or *no*. The value of this node at time *t* depends on values of *Other cancers* and *Death from other cancers* at time *t-1*. It is easy to infer that *P(Death from other cancers*^*(t)*^ = *yes | Other cancers*^*(t-1)*^ = *no)* = *0* and that *P(Death from other cancers*^*(t)*^ = *yes | Death from other cancers*^*(t-1)*^ = *yes)* = *1*. To estimate *P*(*Death from other cancers*^*(t)*^ = *yes | Other cancers*^*(t-1)*^ = *yes, Death from other cancers*^*(t-1)*^ = *no)* we use information from several clinical reports indicating that other cancer is the cause of death in 30% of all patients with CLL within 25 years from diagnosis. Then, we used Eq. (3) to calculate probability of this event in each 6-month period^48^.

3 $${P}_{k}\left(X\right)=-\frac{k}{m}\mathrm{ln}(1-{P}_{m}\left(X\right))$$

where *k* is a unit time period (i.e. 6 months), *m* is the time interval for which the probability of *X* is known.

Using Eq. (3) for the *Death from other cancers* node we have: *k* = 6 months, *m* = 25 × 6 months = 150 months, *P*_*m*_*(X)* = *P*_*150*_*(Death from other cancers* = *yes)* = 0.3 and *P*_*6*_*(Death from other cancers* = *yes)* = 0.007. Consequently, *P*(*Death from other cancers*^*(t)*^ = *no | Other cancers*^*(t-1)*^ = *yes, Death from other cancers*^*(t-1)*^ = *no)* = *0.993*. In nodes with more predecessors, where the available domain literature data is not detailed enough, probabilities were estimated based on expert’s answers to questions concerning particular nodes, e.g. how many times more often *Infections* occurs in patients with *Autoimmune complications* or how many times *Autoimmune complications* reduce probability of curing *Infections* in 6 month period, etc. Finally, each node is present in the network because we are interested in its value and/or, according to the literature or experts, its value influences values of other nodes and/or it is relevant for survival of patients with CLL. For example, *Sex* node was included in HSN network because of a few reasons. Firstly, because incidence of CLL is different in males and females. Secondly, because the patient can die due to a few causes represented by a few nodes in the network. One of these nodes is *Death from other causes*. Because the life expectancy is different in female than in male, it is necessary to include the *Sex* node in the network to capture this relationship and differentiate conditional probabilities in the *Death from other causes* between two sexes. Similar reasoning justifies all other nodes in both networks presented in the “Results” section.

## Data Availability

Data will be made available from the corresponding author under reasonable request.
